# Supplementary material for: Blood cell ratio biomarkers of systemic inflammation in chronic obstructive pulmonary disease
Source: Respir Res. 2026 Mar 18;27:184. doi: 10.1186/s12931-026-03632-3 (PMC13113035; doi:10.1186/s12931-026-03632-3)

Blood Cell Ratio Biomarkers of Systemic Inflammation in Chronic Obstructive Pulmonary Disease

Kaman So, Aabida Saferali, Jeong H. Yun, Min Hyung Ryu, Enrico Schiavi, Pater J. Castaldi, Lisa Ruvuna, Russell P. Bowler, Jeffrey L. Curtis, Craig P. Hersh

Online Supplement

Supplemental Table 1: Quartile cutoffs for ROC curve analysis of 2 or more exacerbations in the prior year

|  | Quartile | value | specificity | sensitivity | AUC |
| --- | --- | --- | --- | --- | --- |
| NLR, all subjects | 1st | 1.50 | 74.50 | 72.10 | 78.99 |
|  | median | 2.08 | 76.80 | 70.37 | 79.12 |
|  | 3rd | 2.87 | 69.14 | 78.52 | 79.33 |
| NLR, COPD GOLD 2-4 | 1st | 1.73 | 72.06 | 65.94 | 74.82 |
|  | median | 2.41 | 76.81 | 61.23 | 75.05 |
|  | 3rd | 3.33 | 78.68 | 58.70 | 75.06 |
| PLR, all subjects | 1st | 92.35 | 73.16 | 73.33 | 78.95 |
|  | median | 119.76 | 74.83 | 71.11 | 79.10 |
|  | 3rd | 154.78 | 75.32 | 70.62 | 79.05 |
| PLR, COPD GOLD 2-4 | 1st | 95.47 | 74.55 | 63.41 | 74.88 |
|  | median | 125.83 | 74.63 | 63.04 | 74.84 |
|  | 3rd | 164.31 | 68.23 | 70.29 | 74.99 |
| SII, all subjects | 1st | 331.10 | 76.20 | 70.12 | 79.02 |
|  | median | 479.50 | 67.86 | 78.52 | 79.28 |
|  | 3rd | 699.30 | 72.64 | 75.80 | 79.69 |
| SII, COPD GOLD 2-4 | 1st | 375.80 | 76.81 | 61.23 | 74.96 |
|  | median | 548.30 | 67.21 | 71.01 | 75.24 |
|  | 3rd | 822.20 | 66.28 | 70.65 | 75.56 |

Tables in separate excel files

Supplemental Tables 2-7: Differential expression of genes and proteins

Supplemental Tables 8-10: RNA-sequencing pathway analyses

Supplemental Tables 11-13: Proteomics pathway analyses

Supplemental Figure 1. Receiver operating characteristic curves for frequent exacerbations outcome (2 or more in the prior year), limited to subjects with COPD GOLD 2-4.


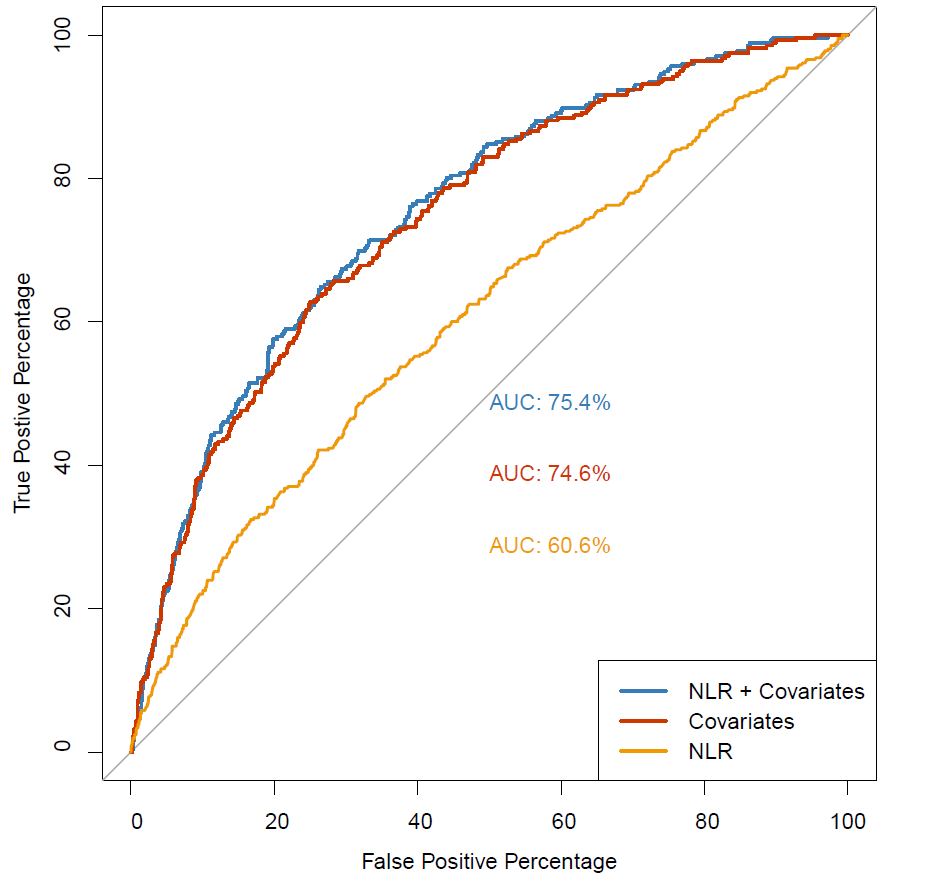

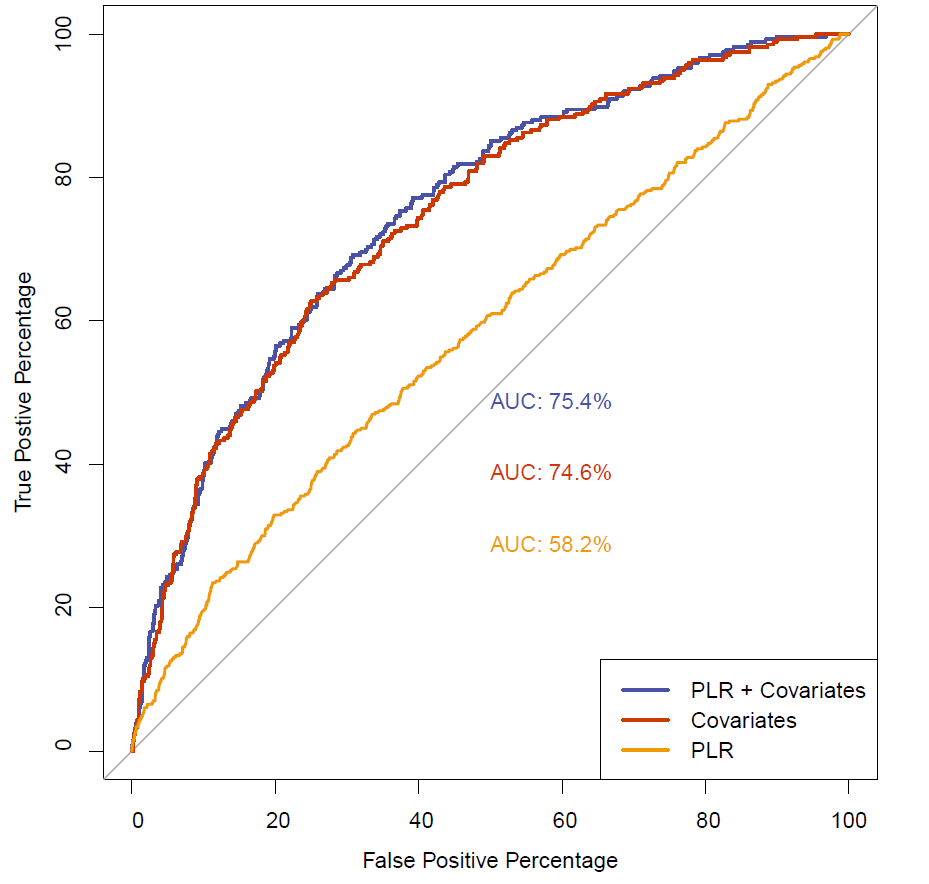

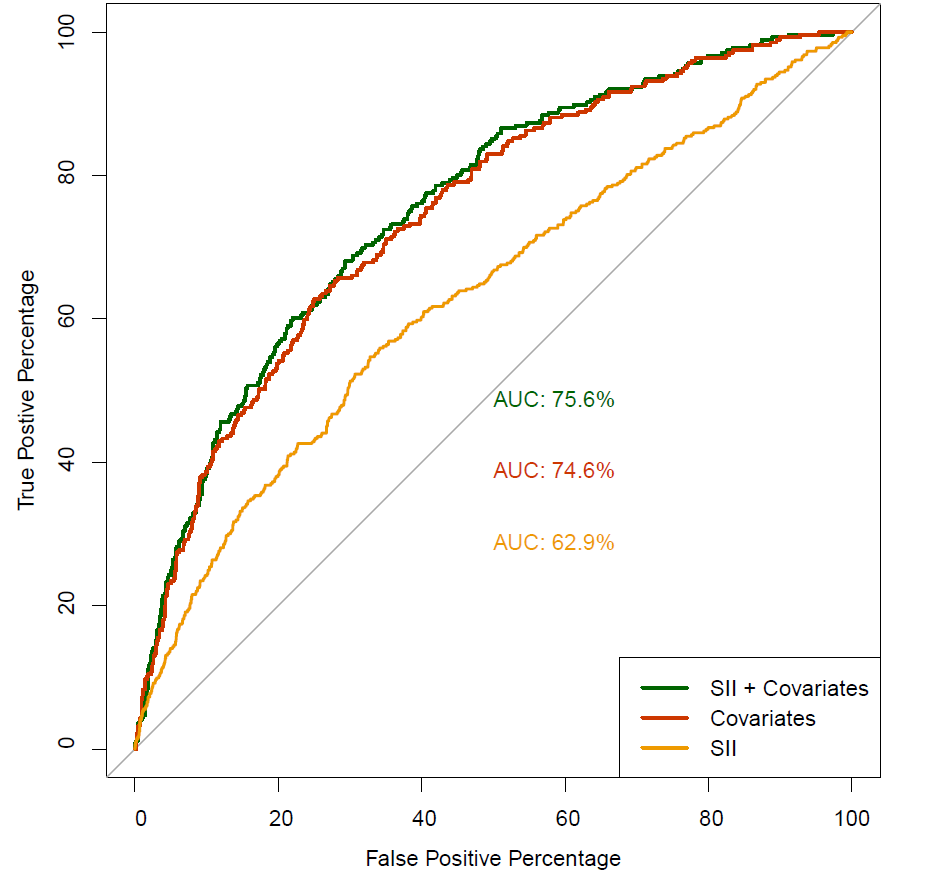


Supplemental Figure 2. Volcano plots of RNA-sequencing. Full results are listed in Supplemental Tables 2-4. Log2 fold change is the magnitude and direction of the association between genes expression and the quantitative blood cell biomarkers.

Supplemental Figure 3. Volcano plots of proteomics. Full results are listed in Supplemental Tables 5-7. Log2 fold change is the magnitude and direction of the association between protein level and the quantitative blood cell biomarkers.


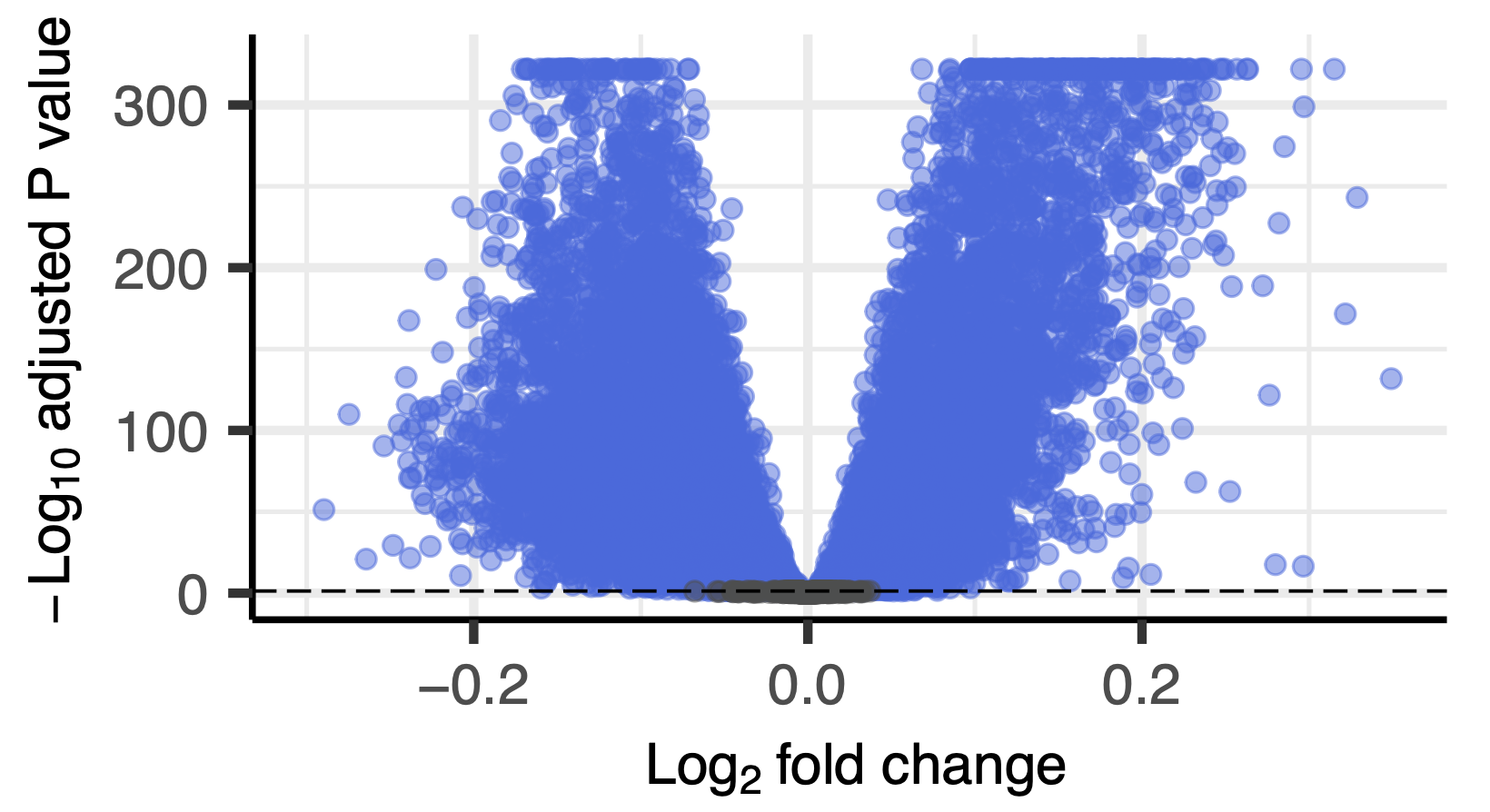

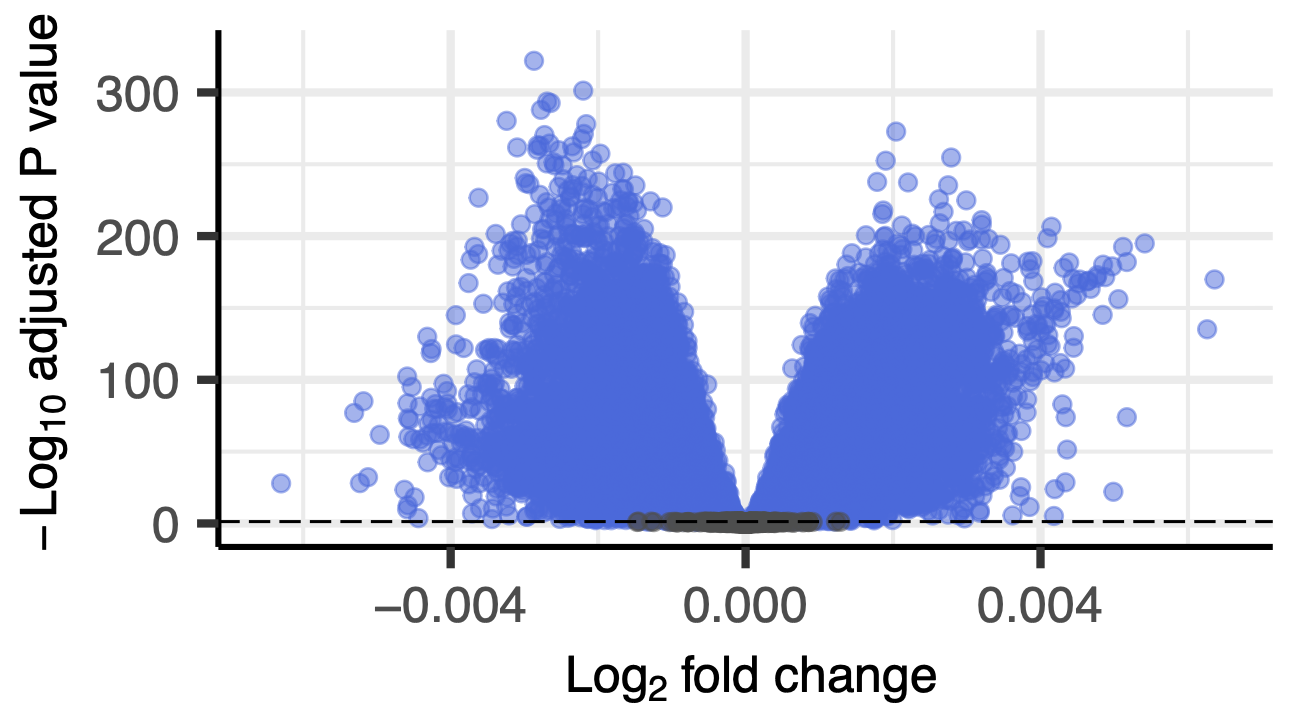


PLR


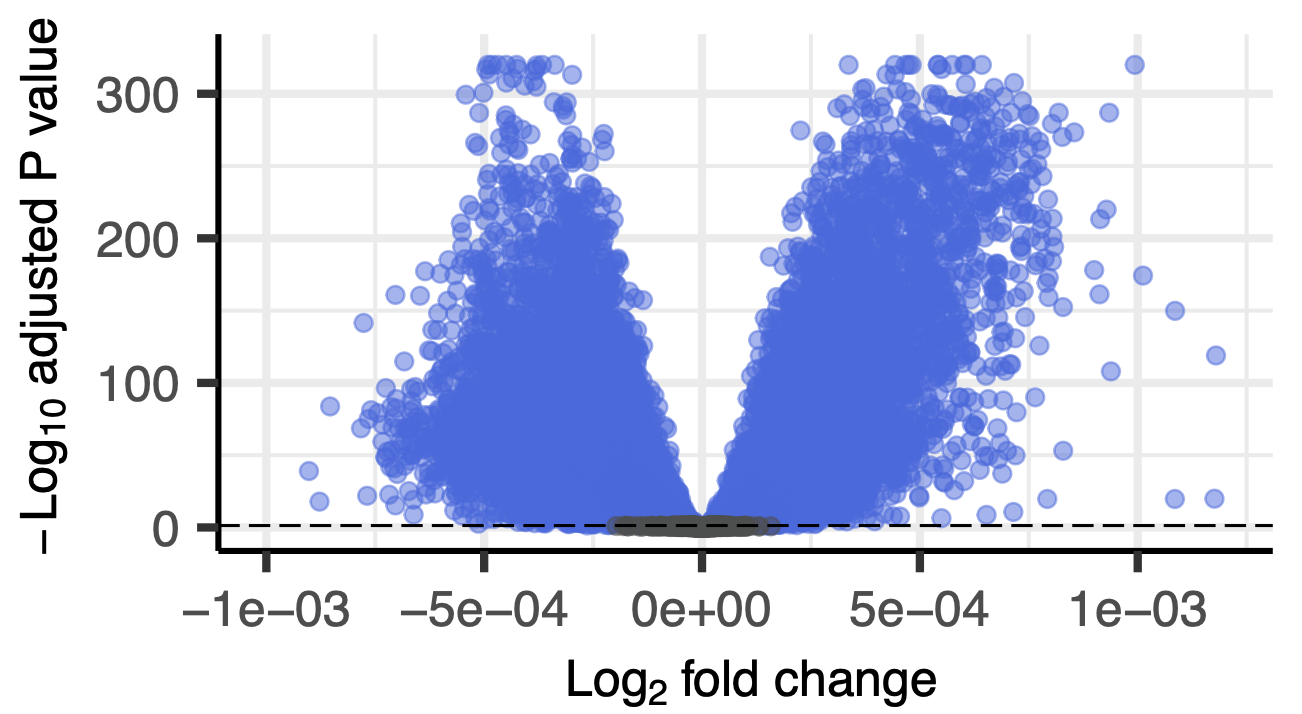


SII

NLR

NLR


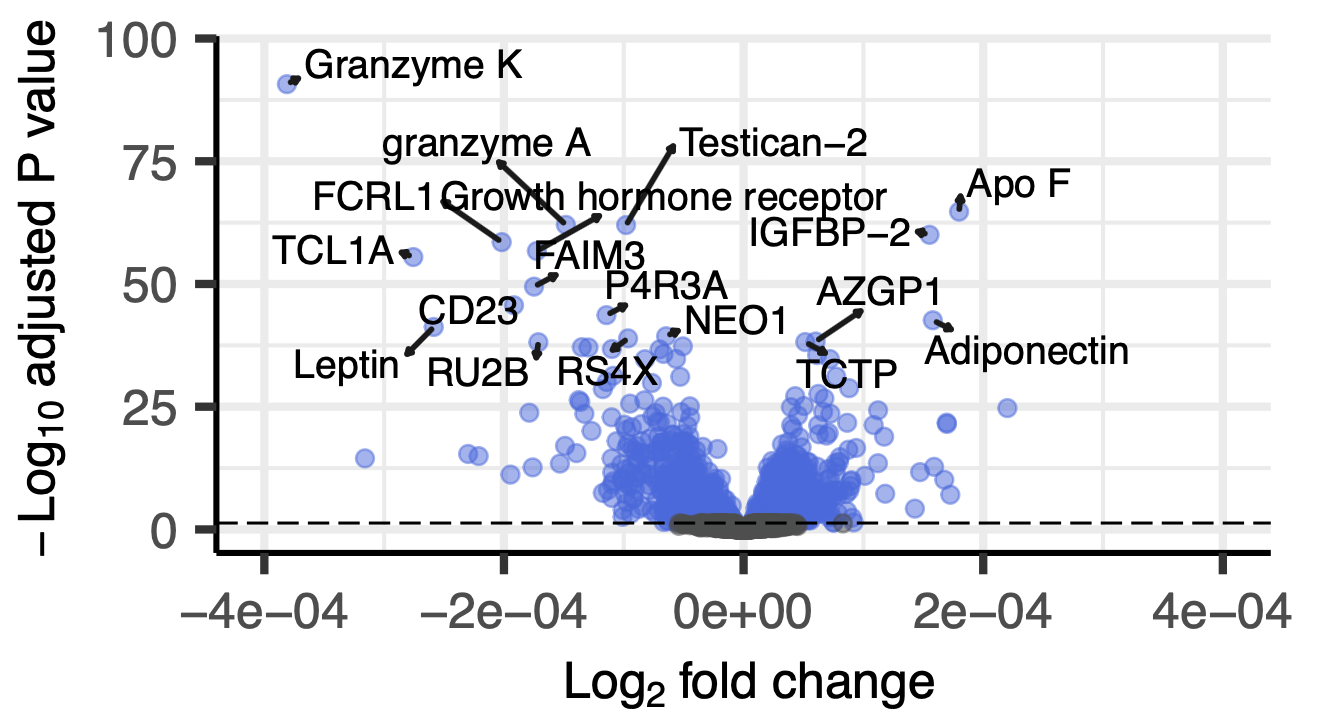


PLR

SII


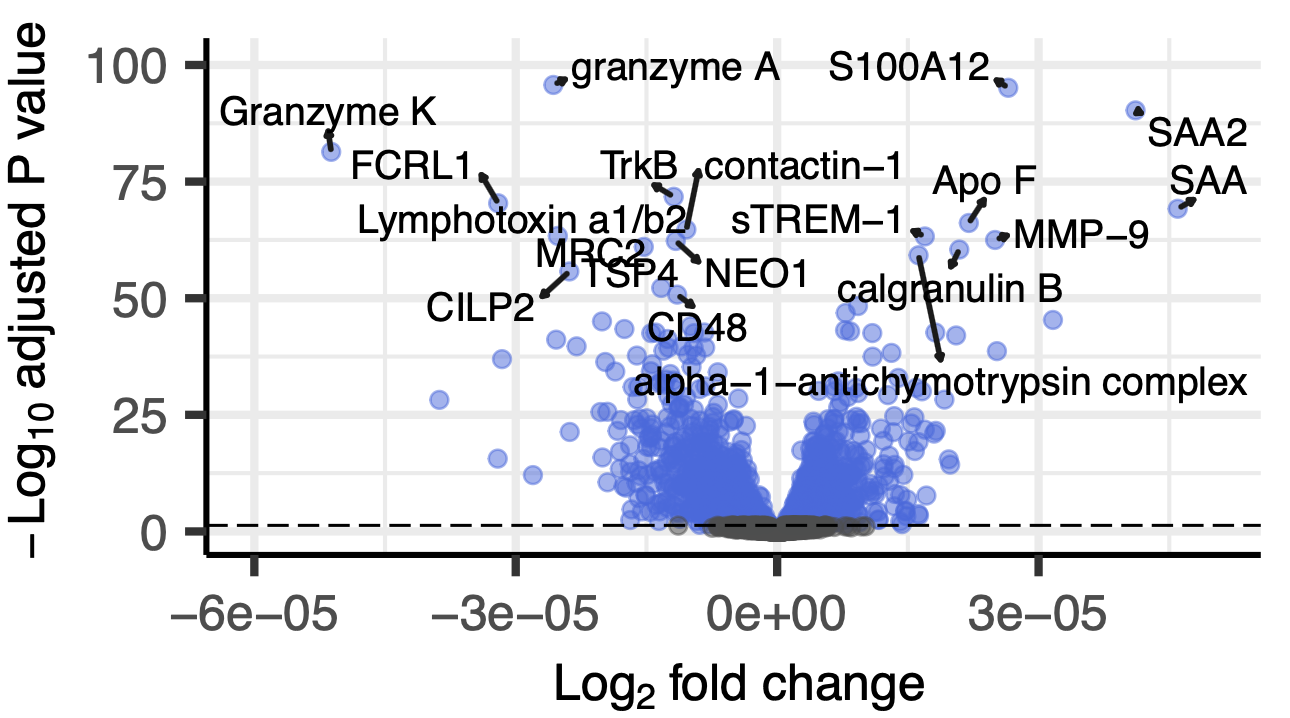

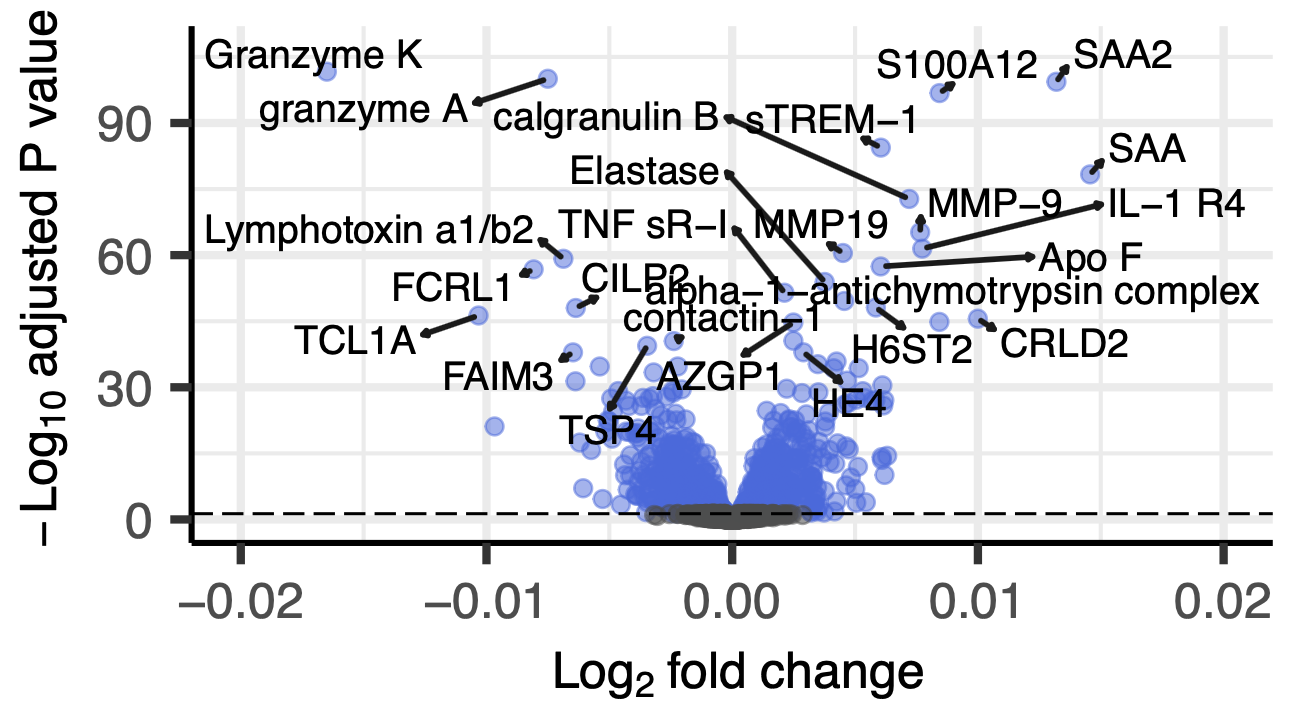

Supplement: Supplementary file 1 — Supplementary Material 1. [file 12931_2026_3632_MOESM1_ESM.docx]
